# Comparative Proteomic Analysis of *saccharopolyspora spinosa *SP06081 and PR2 strains reveals the differentially expressed proteins correlated with the increase of spinosad yield

**DOI:** 10.1186/1477-5956-9-40

**Published:** 2011-07-16

**Authors:** Yushuang Luo, Xuezhi Ding, Liqiu Xia, Fan Huang, Wenping Li, Shaoya Huang, Ying Tang, Yunjun Sun

**Affiliations:** 1 Hunan Provincial Key Laboratory of Microbial Molecular Biology--State Key Laboratory of Breeding Base of Microbial Molecular Biology, College of Life Science, Hunan Normal University, Changsha 410081, P. R. China

## Abstract

**Background:**

*Saccharopolyspora spinosa *produces the environment-friendly biopesticide spinosad, a mixture of two polyketide-derived macrolide active ingredients called spinosyns A and D. Therefore considerable interest is in the improvement of spinosad production because of its low yield in wild-type *S. spinosa*. Recently, a spinosad-hyperproducing PR2 strain with stable heredity was obtained from protoplast regeneration of the wild-type *S. spinosa *SP06081 strain. A comparative proteomic analysis was performed on the two strains during the first rapid growth phase (RG1) in seed medium (SM) by using label-free quantitative proteomics to investigate the underlying mechanism leading to the enhancement of spinosad yield.

**Results:**

In total, 224 proteins from the SP06081 strain and 204 proteins from the PR2 strain were unambiguously identified by liquid chromatography-tandem mass spectrometry analysis, sharing 140 proteins. A total of 12 proteins directly related to spinosad biosynthesis were identified from the two strains in RG1. Comparative analysis of the shared proteins revealed that approximately 31% of them changed their abundance significantly and fell in all of the functional groups, such as tricarboxylic acid cycles, glycolysis, biosynthetic processes, catabolic processes, transcription, translation, oxidation and reduction. Several key enzymes involved in the synthesis of primary metabolic intermediates used as precursors for spinosad production, energy supply, polyketide chain assembly, deoxysugar methylation, and antioxidative stress were differentially expressed in the same pattern of facilitating spinosad production by the PR2 strain. Real-time reverse transcriptase polymerase chain reaction analysis revealed that four of five selected genes showed a positive correlation between changes at the translational and transcriptional expression level, which further confirmed the proteomic analysis.

**Conclusions:**

The present study is the first comprehensive and comparative proteome analysis of *S. spinosa *strains. Our results highlight the differentially expressed proteins between the two *S. spinosa *strains and provide some clues to understand the molecular and metabolic mechanisms that could lead to the increased spinosad production yield.

## Background

The soil actinomycete *Saccharopolyspora spinosa *produces secondary metabolites called spinosyns, polyketide-derived macrolide active ingredients in a family of insect control agents [1]. Chemically, spinosyns are composed of a 21-carbon tetracyclic macrolide containing forosamine and tri-*O*-methyl rhamnose with different degrees of methylation on the polyketide or deoxysugars. The most active and abundant components of the spinosyn family of compounds are spinosyns A and D (spinosad) [2]. The biosynthetic pathway of spinosad involves 23 genes; except for four genes involved in rhamnose biosynthesis, all other genes are grouped in the 74 kb *spn *cluster, including five genes encoding a large polyketide synthetase, four genes for cross-bridging the polyketide lactone, five genes for forosamine biosynthesis, three *O*-methyltransferase genes for rhamnose synthesis and modification, and two glycotransferase genes [3,4]. Spinosad is widely used in agriculture as a potent insect control agent and animal health product because of its high efficiency against target insects and environment-friendly characteristics [5-7].

Spinosad is generally synthesized at very low levels in wild-type *S. spinosa*. Therefore, rational genetic or metabolic engineering strategies for enhancing spinosad yield have attracted much attention. Despite much investigation to improve spinosad production [8-12], little is known about the molecular basis for increasing spinosad production. Recently, a spinosad-producing *S. spinosa *SP06081 strain (CCTCC: M208034) was isolated from a soil sample collected from a sugarcane habitat in Hunan Province, China. Subculture and shake flask fermentation experiments indicated that SP06081 is genetically stable and showed higher spinosad yields than that of the *S. spinosa *DSM 44228^T ^strain. Further protoplast preparation and regeneration was applied to the SP06081 strain [12], thereby producing a spinosad-hyperproducing PR2 strain with stable heredity. The PR2 strain exhibited not only higher productivity of spinosad but stronger resistance to hypoxia than original strain. A comparative proteomic analysis was performed on SP06081 and PR2 strains during the first rapid growth phase (RG1) in seed medium (SM) using label-free quantitative proteomics based on the exponentially modified protein abundance index (emPAI) [13-15] to investigate the molecular and metabolic mechanisms underlying the improvement in spinosad production. Primarily, we studied the correlation between the differential expression of proteins during RG1 in SM and the spinosad yield increase in the *S. spinosa *strain and identified some particular proteins of interest as targets for rational manipulation of the industrially important strain to increase spinosad yield.

## Results

### Physiologic Characteristics of *S. spinosa *SP06081 and PR2

**Figure 1:**
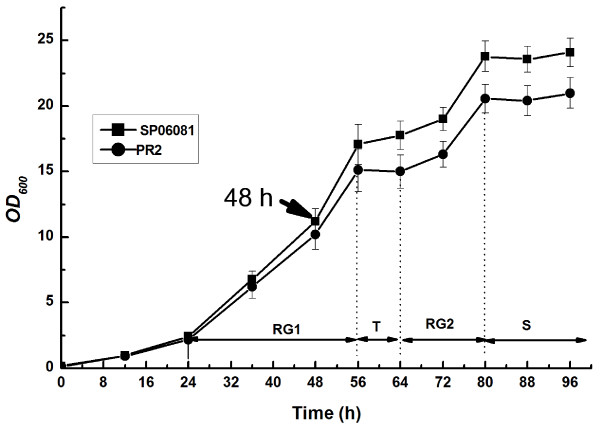
**Growth-time curve of *Saccharopolyspora spinosa *SP06081 and PR2 strains in SM**. RG, rapid growth phase; T, transition phase; S, stationary phase. Arrow indicates the time point chosen for biomass collection used for shotgun protein sample preparation and RNA isolation. Three replicates were performed for each strain. Error bars indicate standard error of the mean.

*S. spinosa *SP06081 and PR2 incubated in SM showed reproducible biphasic growth kinetics (Figure 1), with a first rapid growth, RG1 (24-56 h), a transition phase (56-64 h), a second rapid growth (64-80 h), and a stationary phase (after 80 h). Although no difference was observed in morphology between the two strains, the growth rate of the PR2 strain exhibited a slight decrease compared with that of the SP06081 strain during the same culture period.

The different capacity of spinosad production in different media and oxygen supply conditions was observed between the two strains (see Additional file 1: Figs. S1 and S2).

### Identification of the Total Proteome

**Figure 2:**
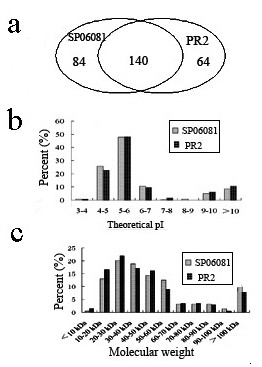
**(a-c) Distribution of the identified proteins by LC-MS/MS**. The distribution of total proteome showed (a) the overlap of proteins between SP06081 and PR2 strains, (b) the theoretical pI distribution of the total proteome, and (c) the Mw distribution of the total proteome.

Proteins extracted from the two strains were digested with trypsin, and the resultant peptide mixtures were independently analyzed three times by liquid chromatography-tandem mass spectrometry (LC-MS/MS). A more than 90% concordance in the repertoire of identified proteins was obtained among the three independent LC-MS/MS analyses of each sample. LC-MS/MS experiments of the two strains resulted in a pool of 288 unique protein identifications when combined; 224 and 204 proteins were unambiguously identified from the SP06081 and PR2 strains (see Additional file 2 and file 3: Tables S2 and S3), respectively, sharing 140 proteins (Figure 2a). The distribution of the theoretical isoelectric point (pI) as well as the molecular weight (Mw) of the identified proteins showed a very similar pattern in the two strains. In detail, the pI distribution, ranging from 3.66 to 12.01, was bimodal, located mainly in the ranges pI 4.0-7.0 and 9.0-10.0, and only approximately 1.4% of proteins with pI between 7.0 and 9.0 were identified (Figure 2b), whereas the Mw distribution, ranging from 10 to 100 kDa, was located mainly in the ranges Mw 10-60 kDa (Figure 2c). Proteins with extreme pI and Mw were also detected. Approximately 10% of proteins with pI > 10.0 and 1% of proteins with pI < 4.5 were identified, whereas approximately 10% of proteins with Mw > 100 kDa and 0.5%-1% of proteins with Mw < 10 kDa were observed (Figures 2b and 2c).

### Gene Ontology Analysis of the Identified Proteins

**Figure 3:**
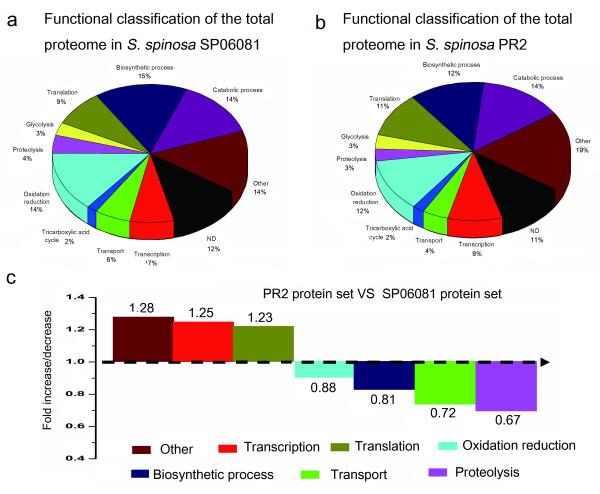
**Fold changes of functional categories of the total proteome between *Saccharopolyspora spinosa *SP06081 and PR2 strains**. The total proteomes of (a) SP06081 and (b) PR2 strain protein set were classified into functional categories, and (c) the fold change of the different functional classes between these two sets was calculated as follows: the percentage of a specific category of the PR2 strain protein set divided by the percentage of that category in the SP06081 strain.

**Figure 4:**
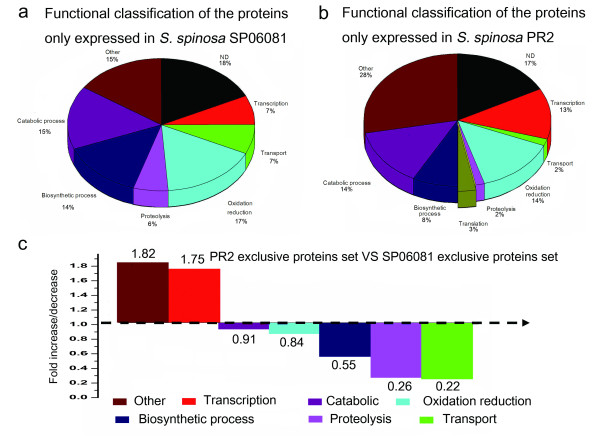
**Fold changes of functional categories of the exclusive proteins between *Saccharopolyspora spinosa *SP06081 and PR2 strains**. The proteins only expressed in (a) SP06081 and (b) PR2 strain were classified into functional categories, and (c) the fold change of the different functional classes between these two sets was calculated as follows: the percentage of a specific category of the PR2 strain protein set divided by the percentage of that category in the SP06081 strain.

Total proteins identified from the two strains were categorized into gene ontology (GO) classes (see Additional file 2 and file 3: Tables S2 and S3) and comparatively analyzed to study the changes in the global gene expression profile. Among the identified proteins, approximately 90% were assigned as having a predicted function and classified into 10 functional categories according to their biological processes (Figures 3a and 3b). For the SP06081 strain, of 224 nonredundant proteins, a large number of identified proteins were related to biosynthetic processes (15%) as well as to catabolic processes (14%) and oxidation reduction (14%). The remainder were involved in transcription (7%), transport (6%), tricarboxylic acid (TCA) cycle (2%), proteolysis (4%), glycolysis (3%), translation (9%), and other annotated proteins (14%), with special molecular functions such as protein folding and DNA repair (Figure 3a). We then compared the proportion (in percentage) of the functional categories of the total proteome and exclusive protein set from the PR2 strain (Figures 3b and 4b) with those from the SP06081 strain (Figures 3a and 4a) and calculated the fold change (increase/decrease) between these two sets (Figures 3c and 4c). The calculation was done as follows: the percentage of a specific category of the PR2 protein set divided by the percentage of that category in the SP06081 proteome. Hence, a fold-change value > 1 indicates an increase of a particular category in the PR2 protein set, and vice versa. For the total proteome, all functional categories except catabolic processes, glycolysis, and the TCA cycle showed fold changes between the two strains. Translation, transcription, and other categories were increased in the PR2 strain, whereas all other functional categories were decreased (Figure 3c). In terms of exclusive proteins, the fold change in functional categories (Figure 4c) showed a similar trend with that of the total proteome (Figure 3c).

### Semiquantitative Comparative Analysis of the LC-MS/MS Identified Proteins

The abundance of the identified proteins was calculated using emPAI values to investigate the differential expression of these proteins between the two strains. 84 and 64 proteins were exclusively identified from the SP06081 and PR2 strains, respectively (see Additional file 4: Tables S4 and S5). Among these proteins, the top five proteins with a high emPAI value from the SP06081 strain were PTn (a putative transposase), PNP (an enzyme involved in purine metabolism), PTCSRR (a protein that function as a receptor in a two-component signal transduction regulatory system), H/Y/Y (a Fe-S protein), and HP2348 (a protein of unknown function), whereas the top five proteins from the PR2 strain were MASPP (an enzyme involved in signal transduction), TR1 (a protein of unknown function), CSDBDP (a cold-shock protein of the CspA family), NDSI (an enzyme involved in oxidative stress), and SP (a protein of unknown function).

**Figure 5:**
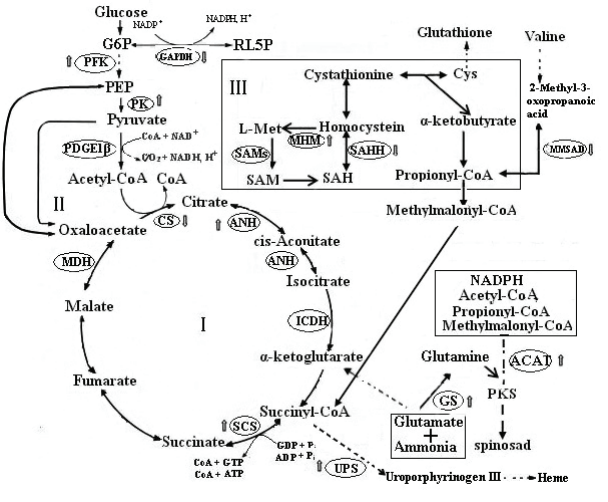
**Integrated proteome data onto a simplified metabolic network**. I: TCA cycle; II: Glycolysis;III: SAM degradation pathway. Pathways and corresponding enzymes were identified from Swiss-Prot and TrEMBL databases, and only major reactions in each pathway are shown. Protein names are provided in Additional file 5: Table S6. Up or down arrows show the up- or down-regulation of proteins in PR2 strain, respectively. Abbreviations: PDGE1β, pyruvate dehydrogenase E1 component beta subunit; PEP, phosphoenolpyruvate; G6P, glucose-6-phosphate; RL5P, ribulose 5-phosphate; SAH, *S*-adenosyl-homocysteine; PFK, 6-phosphofructokinase; PK, pyruvate kinase; GAPDH, glyceraldehyde 3-phosphate dehydrogenase; MMSAD, methylmalonate semialdehyde dehydrogenase (acylating); GS, glutamine synthetase; CS, citrate synthase; ACAT, acetyl-CoA acetyltransferase; MHM, 5-methyltetrahydropteroyltriglutamate-homocysteine methyltransferase; SCS, succinyl-CoA synthetase subunit alpha; UPS, uroporphyrinogen III synthetase; SAM, *S*-adenosylmethionine; PKS, polyketide synthase; L-Met, L-methionine; CoA, coenzyme A; NAD^+^, nicotinamide adenine dinucleotide; NADH, reduced form of nicotinamide adenine dinucleotide; NADP^+^, nicotinamide adenine dinucleotide phosphate; NADPH, reduced form of nicotinamide adenine dinucleotide phosphate.

The relative abundance of 140 shared proteins was then compared using their emPAI values to extend the comparative analysis to those proteins shared by the two strains. Most of them (~69%) showed similar expression levels. Conversely, 21 proteins increased and 23 proteins decreased their abundance in a statistically significant manner in the PR2 strain and fell in all of the functional groups (see Additional file 5: Table S6). Proteomics data were plotted on a simplified metabolic reaction network using gene annotations taken from Swiss-Prot and TrEMBL databases (Figure 5) to further elucidate the molecular mechanism underlying the improvement of spinosad production. Interestingly, in the PR2 strain, several proteins (6-phosphofructokinase [PFK], pyruvate kinase [PK], glyceraldehyde 3-phosphate dehydrogenase [GAPDH], and methylmalonate semialdehyde dehydrogenase [MMSAD], 5-methyltetrahydropteroyltriglutamate-homocysteine methyltransferase [MHM], acetyl-CoA acetyltransferase [ACAT]) related to the production of the precursors required for polyketide biosynthesis, such as the reduced form of nicotinamide adenine dinucleotide phosphate (NADPH), acetyl-coenzyme A (CoA), and propionyl-CoA, were differentially expressed in the same direction to increase the availability of these compounds. Moreover, two key enzymes (citrate synthase [CS] and succinyl-CoA synthetase subunit alpha [SCS]) involved in TCA cycle exhibited an opposite variation trend. The abundance of CS decreased more than 3-fold whereas that of SCS increased 1.9-fold in the PR2 strain compared with the SP06081 strain. Furthermore, differential expressions of proteins were observed in several proteins involved in response to oxidative stress (alkyl hydroperoxide reductase [AhpC]/thiol-specific antioxidant [TSA], uroporphyrinogen III synthetase [UPS] and GAPDH) as well as in the proteins related to signal transduction (ABC transporter ATP-binding protein [ABP]) and the regulation of secondary metabolism (glutamine synthetase [GS]).

Notably, a total of 12 proteins directly related to spinosad biosynthesis were identified from the two strains during RG1 in SM, 9 of these proteins participate in polyketide synthesis, 2 proteins in forosamine synthesis, and 1 protein in rhamnose synthesis (Table 1). All of these proteins have a low abundance with the exception of acyl carrier protein (ACP). Moreover, 3 proteins (megalomicin 6-deoxy-erythronolide B synthase 1 [M6DBS1], putative NDP-3-keto-6-deoxyhexose 3-ketoreductase [PNDP-DK] and BusQ) and 4 proteins (polyketide synthase extender modules 8-10 [PSEM8-10], polyketide synthase extender module 2 [PSEM2], 6-deoxyerythronolide B synthase II [6DBSII] and TDP-4-keto-6-deoxyhexose 3,5-epimerase [TDP-DE]) were exclusively identified from the PR2 and SP6081 strains, respectively, while five proteins with nearly the same protein abundance were identified from the both strains (Table 1).

**Table 1:** Summary of the identified proteins directly related to spinosad biosynthesis pathway from *Saccharopolyspora spinosa *SP06081 and PR2 strains during RG1 in SM

Accession number ^1)^	Protein description	Gene ^2)^	Possible function ^3)^	emPAI value^4)^		Sequence coverage (%)		SEQUEST Score ^5)^	
				
				PR2	SP06081	PR2	SP06081	PR2	SP06081
gi134098110	acyl carrier protein	ACP	Polyketide synthesis	0.93	0.93	13	13	40	48
gi134100839	modular polyketide synthase	MPS	Polyketide synthesis	0.01	0.01	5	5	48	35
gi30795006	lankamycin synthase, modules 3 and 4	LSM3-4	Polyketide synthesis	0.013	0.018	2	1	70	63
gi30795005	lankamycin synthase, modules 5 and 6	LSM5-6	Polyketide synthesis	0.007	0.007	2	1	55	62
gi10179853	megalomicin 6-deoxy- erythronolide B synthase 2	M6DBS2	Polyketide synthesis	0.006	0.006	3	1	57	81
gi10179852	megalomicin 6-deoxy- erythronolide B synthase 1	M6DBS1	Polyketide synthesis	0.01	/	4	/	100	/
gi30795014	putative NDP-3-keto-6- deoxyhexose 3- ketoreductase	PNDP-DK	Forosamine synthesis	0.04	/	10	/	37	/
gi68270876	BusQ	BusQ	Forosamine synthesis	0.04	/	4	/	20	/
gi13162634	polyketide synthase extender modules 8-10	PSEM8-10	Polyketide synthesis	/	0.004	/	1	/	176
gi13162637	polyketide synthase extender module 2	PSEM2	Polyketide synthesis	/	0.02	/	2	/	115
gi581651	6-deoxyerythronolide B synthase II	6DBSII	Polyketide synthesis	/	0.006	/	2	/	32
gi10179846	TDP-4-keto-6-deoxyhexose 3,5-epimerase	TDP-DE	Rhamnose synthesis	/	0.12	/	11	/	26

1) Protein database accession numbers were obtained on NCBI database.

2) Gene product acronym

3) Functional clustering realized according to Gene Ontology classification http://www.uniprot.org/ and hypothetical pathway for the biosynthesis of spinosyn (see reference 4).

4) The exponentially modified protein abundance index value (emPAI) is the transformed ratio of the number of experimentally observed peptides to the total number of peptides obtained after *in silico *trypsinization of the protein by using IPEP online proteolysis http://ipep.moffitt.org/searchProtein.cgi.

5) The Score is a sum of the individual peptide scores from all six proteomics run (see Additional file 2 and file 3: Tables S2 and S3).

### Real-Time Reverse Transcriptase Polymerase Chain Reaction of Selected Genes

**Figure 6:**
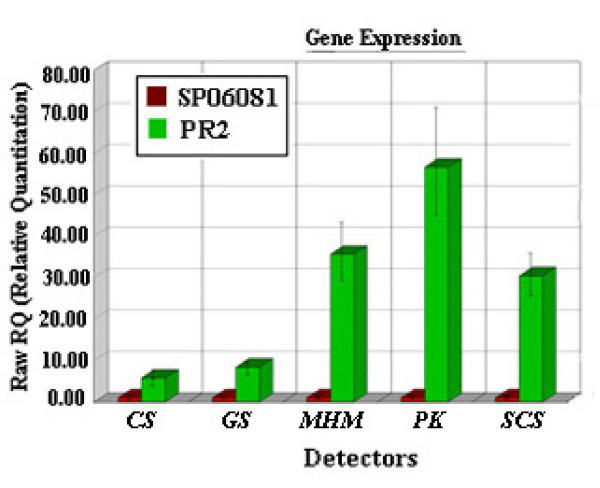
**Real-time RT-PCR analysis of selected genes**. Quantitative real-time RT-PCR was used to substantiate differential expression patterns of five selected genes (*CS, GS, MHM, SCS*, and *PK*). mRNA levels after 48 h culture are expressed as relative values to *sigA*, arbitrarily setting the ratio values for the SP06081 sample to 1. Error bars are calculated from six independent determinations of mRNA abundance in each sample (see Methods section).

Five genes of differentially expressed proteins were selected to measure their relative mRNA levels by real-time reverse transcriptase polymerase chain reaction (RT-PCR) to confirm the proteomic analysis. The products of the selected genes were involved in several metabolic functions as follows: (i) TCA cycle (CS and SCS), (ii) amino acid biosynthesis involved in SAM metabolic pathway (MHM), (iii) glycolysis (PK), and (iv) glutamine biosynthesis (GS). Results showed that four (*SCS, PK, MHM*, and *GS*) of the five selected genes showed up-regulations at the transcriptional level in the PR2 strain as compared with the SP06081 strain, consistent with the results of protein expression (Figure 6, Additional file 5: Table S6), although the mRNA of these genes appeared to be more dynamic with more than eightfold changes and the protein levels changed by less than threefold. However, an inconsistency between the translational and transcriptional level was seen in CS. The mRNA expression of CS showed about a fivefold increase (Figure 6) and the protein abundance exhibited more than a threefold decrease in the PR2 strain compared with the SP06081 strain (see Additional file 5: Table S6).

## Discussion

Previous studies have demonstrated that secondary metabolism is intrinsically linked to the primary metabolism via the supply of precursors and cofactors [16,17]. Recent research has indicated that improved precursor and energy supplies can facilitate antibiotic production [18-21]. The present article demonstrates a correlation between those proteins directly related to the synthesis of precursors and the increase of spinosad yield by a comparative proteomic analysis of wild-type *S. spinosa *SP06081 strain and its hyperproducing PR2 strain, These data provide some clues to understand the molecular and metabolic mechanisms that could lead to the increased spinosad production yield. In previous experiments, inoculum age was observed to have a significant effect on spinosad yield, and the optimal inoculum age was 48 h at RG1 (unpublished results). Therefore, we preferred the 48 h old seed culture as a biomass sample for proteomic analysis. The same set of biomass sample was used to shotgun protein sample preparation and RNA isolation as well as the inoculation for fermentation of *S. spinosa *strains under different oxygen supply conditions to ensure the reliability and the confidence of comparative analysis about the correlation between different expression of the identified proteins and spinosad production. The high degree of similarity in protein profile between the two strains lends biological confidence to the proteomics data. Real-time RT-PCR analysis of selected genes further supports the proteomic semiquantitative results. The inconsistency between transcription and protein expression level in CS is probably due to the diversity in degradation rates between mRNA and protein, similarly to that already observed in other actinomycetes [22,23].

Many biological processes are intrinsically dynamic, incurring profound changes at both molecular and physiological levels. Systems analysis of such processes incorporating large-scale proteome profiling can be quite revealing. The differentially expressed proteins identified from the two strains fell in all of the functional groups (see Additional file 4 and file 5: Tables S4-S6), which suggested that some specific metabolic pathways are differentially controlled by changes in expression levels of key proteins. The identification of 12 proteins directly related to the spinosad biosynthesis pathway implied the trigger of spinosad biosynthesis during RG1 in SM despite the low protein abundance. Notably, 3 and 4 of these proteins were exclusively identified from PR2 and SP06081 strains, respectively (Table 1), implying the different availability of direct precursor such as polyketide, forosamine and rhamnose between the two strains. The exclusively identified proteins from PR2 strain (M6DBS1, PNDP-DK and BusQ) may be mainly involved in the synthesis of direct precursors thus facilitating the accumulation of these compounds.

Integration of the proteome data with the available functional information on the basis of the GO analysis resulted in the identification of three groups of proteins that were differentially expressed in the same pattern of facilitating spinosad production by the PR2 strain. The first group contains the precursor-producing and energy supply proteins, including CS, SCS, MHM, PK, PFK, MMSAD, GAPDH and ACAT. The key rate-limiting enzyme CS, which irreversibly catalyzed the reaction in TCA cycle [24], showed a significant decrease in abundance, whereas the SCS, which is the only enzyme capable of adenosine-triphosphate (ATP) production via substrate level phosphorylation in the absence of oxygen [25], was observed to up-regulate in the PR2 strain compared with the SP06081 strain. Metabolic intermediates from glycolysis have a different destination in the two strains as it is suggested by the downregulation of CS in the PR2 strain. On the other hand, in the PR2 strain energy production may be ensured by the up-regulation of SCS since succinyl-CoA may be synthesized from metabolic pathways different from TCA, as reported in Figure 5, thereby increasing the precursor (acetyl-CoA) required for spinosad biosynthesis and the energy supply. Consequently, the spinosad production potentiality of the PR2 strain was enhanced. MHM is a critical protein participating in the biosynthetic pathway of SAM (Figure 5). In spinosad biosynthetic processes, the two *N*-methyl groups of forosamine and the three *O*-methyl groups of tri-*O*-methyl rhamnose are all derived from SAM [1]. Thus, the up-regulation of MHM may contribute to spinosad biosynthesis, in agreement with previous reports in which SAM was documented as a positive regulator for secondary metabolism in *Streptomyces *[26-28]. The other proteins (PK, PFK, MMSAD, GAPDH and ACAT) related to the biosynthesis of polyketide precursor were all observed to be differentially expressed in the same direction to increase precursor supply in the PR2 strain (Figure 5). The improvement of precursor and energy supply may be related to the increase of spinosad yield, in agreement with previous results [18-21].

The second group contains the redox balancing proteins (AhpC/TSA, UPS, and GAPDH). The AhpC/TSA family protein, namely, peroxiredoxins in mammalian cells [29], usually plays a role of respiratory antioxidants in bacteria [30]. UPS is a key enzyme involved in the biosynthesis of heme [31], which is a prosthetic group of peroxidase [32]. The inactivation of GAPDH can reroute temporally the metabolic flux from glycolysis to the pentose phosphate pathway, allowing the cell to generate more NADPH for counteracting oxidative stress [33]. The co-up-regulation of AhpC/TSA and UPS together with the down-regulation of GAPDH in the PR2 strain promoted speculation that the potency against hypoxia for *S. spinosa *is positively correlated to the spinosad production. The other five protoplast-regenerated strains and the two ^60^Coγ mutation *S. spinosa *strains with higher spinosad yield than the original SP06081 strain were also detected to corroborate the assumption. All detectors showed a similar tendency in the case of maintained spinosad yield to a certain extent under low dissolved oxygen supply (data not shown) as was observed in the PR2 strain (see Additional file 1: Fig. S2).

The third group contains the proteins related to the regulation of secondary metabolism (GS) and signal transduction (ABP, PTCSRR, and MASPP). GS is a key enzyme of nitrogen metabolism and a major player in the link of nitrogen assimilation to antibiotic production [34]. Recent research has demonstrated it is a positive regulator of antibiotic production in *Streptomyces coelicolor *A3 (2) [35]. The up-regulation of GS may positively affect the spinosad production in PR2 strain. Previous research has shown that physiological differentiation, biomass growth, and secondary metabolite biosynthesis in actinomycete require activation of the ribosome population and intra- or intercellular communication systems [36-39]. The differential expression of ABP, PTCSRR, and MASPP is indicative of differential signal transduction between the two strains.

## Conclusions

This is the first comprehensive and comparative proteome analysis of *S. spinosa *strains using label-free quantitative proteomics. Our results provide interesting hints that the differentially expressed proteins mentioned above are very likely related to the improvement of spinosad production, although their exact molecular role is still unknown. Further investigations are required to establish a causal relationship between these proteins and the increase of spinosad yield.

## Methods

### Strains and Growth Conditions

The strains of *S. spinosa *used in the present study were SP06081 (wild-type strain CCTCC: M208034) and the hyperproducing PR2 strain. Mycelia of the two strains were harvested from culture grown in SM (1% glucose, 3% casein peptone, 1.0% yeast extract, 0.05% potassium dihydrogen phosphate, and 0.2% magnesium sulfate heptahydrate) for 48 h at 30°C and used for shotgun protein sample preparation and RNA isolation, whereas a 2 mL portion of the seed culture was transferred into a 20 mL production medium (10% glucose, 3.2% cotton seed powder, 0.8% maize powder, 0.6% soybean powder, 0.4% calcium carbonate, 0.1% yeast powder, 2.5% soybean oil, and 0.25% trace element) for spinosad production as described previously [12] under different oxygen supply conditions. Fermentation for spinosad production was run for 9 days in a humidified rotary shaker incubator (NBS, USA) at 30°C and 80% relative humidity. Two biological replicates of *S. spinosa *SM cultures were prepared for each strain, whereas three technical replicates were performed for protein analysis, real-time RT-PCR analysis, and spinosad production for each biomass. The collected samples were quickly frozen in liquid nitrogen and kept at -80°C before protein sample preparation and RNA extraction.

### Analysis of Spinosad by High-Pressure Liquid Chromatography

The ÄKTA Purifier10 (GE Healthcare, USA) high-pressure liquid chromatography (HPLC) system was used for the analysis of spinosad yield. A suspension of the culture (1 mL) was extracted with 1 mL of methanol for 10 h at 30°C. Methanol extracts were collected by centrifugation at 12,000 rpm for 30 min at 4°C. After filtration through 0.22 μm Millipore filters, the supernatants (10 μL) were loaded onto a C18 column (AQ12S05-1546WT, 150 × 4.6 mm ID, S-5 μm, 12 nm) then eluted with methanol-acetonitrile (ACN)-2% aqueous ammonium acetate (vol/vol/vol = 45:45:10) at 1.5 mL/min. The detection wavelength was 250 nm.

### Protein Sample Preparation

Mycelia were collected and washed three times with ice-cold phosphate-buffered saline, pelleted by centrifugation at 4°C, and quickly frozen in liquid nitrogen and ground to a fine powder using a prechilled mortar and pestle. The cell powder (0.2 g) was suspended in 0.8 mL lysis buffer composed of 8 M urea, 4% (wt/vol) CHAPS, 40 mM Tris, 65 mM dithiothreitol (DTT), and 5 μL protease inhibitor cocktail in a sterilized Eppendorf tube at 4°C for 1 h. After centrifugation at 13,200 rpm for 30 min, the supernatant protein extract was stored at -80°C. Before proteolysis, the protein sample was purified by using Cleanup kit, and the protein content was quantified by using Quant kit. Then, a 50 μg portion of the total protein was subjected to in-solution digestion with trypsin at a ratio of 1:50 (wt/wt), denatured with 8 M urea for 1 h, reduced with 5 mM DTT, alkylated with 25 mM iodoacetamide, digested 16 h at 37°C, and then the digestion was terminated with formic acid.

### LC-MS/MS Analysis of Peptides

LC-MS/MS experiments were performed on an LTQ XL hybrid mass spectrometer (Thermo Fisher Scientific, USA) coupled to a Finnigan LC system (Thermo Fisher Scientific). Peptide mixtures were loaded and desalted online in a reverse-phase precolumn (C18 Pepmap column, LC Packings) and resolved on a nanoscale C18 Pepmap TM capillary column (LC Packings) at a flow rate of 500 nL/min with a gradient of ACN/0.1% FA before injection into the ion trap mass spectrometer. Peptides were separated using a 65 min linear gradient from 0% to 100% ACN in 0.1% FA. Spectra were obtained in a data-dependent acquisition mode, which consisted of a survey scan over the *m/z *range of 300-2000 followed by five scans on the most intense precursor ions activated for 30 ms by the excitation energy level of 35%. The zoom scan function was set off, and dynamic exclusion was applied.

### Protein Identification

With Xcalibur Software (Thermo Finnigan, Palo Alto, CA) and the SEQUEST algorithm, the raw MS/MS data were searched against the nonredundant protein database (updated November 20, 2009), downloaded as FASTA formatted sequences from the National Center for Biotechnology Information Web site (http://www.ncbi.nlm.nih.gov/protein). Search parameters were set as follows: taxonomy, *Saccharopolyspora*; enzyme, trypsin; allowing up to one missed cleavage; peptide mass tolerance was 2.5 Da, MS/MS mass tolerance was 0.8 Da, and fixed modification parameter was carbamidomethylation. We applied the target-decoy search strategy by searching the MS/MS spectra against the reversed and randomized *Saccharopolyspora *proteome sequences to assess the rate of false-positive identifications. All of the peptide matches were filtered on the basis of their false-positive rate (<1%) [40]. The lowest Xcorr values of the peptide were set to 1.9 (+1 charge), 2.2 (+2 charge), and 3.75 (+3 charge), and the uniqueness scores of matches were (delta Cn) > 0.1. Proteins with more than two unique peptides or a single unique peptide that has at least seven amino acids and had a high-quality spectra containing at least three consecutive b- or y-ions were considered as reliably identified.

### Bioinformatics Annotation and Protein Classification

The function of the identified proteins was elucidated by GO classification (http://www.uniprot.org/) against the Swiss-Prot and TrEMBL databases (*Saccharopolyspora *taxonomy); text-based annotation files were available for download from the GO database ftp site ftp://ftp.geneontology.org/pub/go/[41]. Unclassified proteins were placed in the "no description" category. The proportion (in percentage) of the functional categories from PR2 protein set was compared with those from the SP06081 proteome by calculating the fold change (increase/decrease) between these two sets to investigate the differences in protein composition concerning molecular processes between the two strains.

### Semiquantitative Analysis of Proteins

Protein abundance was determined by semiquantitative analysis as described earlier [13-15]. Briefly, the total spectral count (SC) of MS/MS spectra for each detected protein was determined and then normalized by dividing the number of counted spectra through the number of predicted observable peptides (OP) to correct the determined SC for differences in protein size. The OP was obtained after in silico trypsinization of the protein by using the IPEP online proteolysis (http://ipep.moffitt.org/searchProtein.cgi) [42]. Not all theoretical tryptic peptides can be identified because of some technical restrictions; hence, the peptides beyond the scan range of the LTQ mass spectrometer (600-3500 Da) were eliminated. Protein abundance estimates were obtained from emPAI, calculated as follows:

The differential analysis was carried out on the biomasses collected from two parallel cultures for each strain. For each biomass, three technical replicates were performed for LC-MS/MS and statistical analysis. Thus, protein quantification was calculated as mean emPAI (MEP) by using data from six shotgun proteome. Mean square deviation (MSD) was also calculated for statistical validation. Therefore, the expression of each protein was defined as an interval whose upper and lower limits were calculated as [MEP + MSD] and [MEP - MSD], respectively. Differential analysis was evaluated statistically (*t*-test), and increased (I) or decreased (D) protein abundance was accepted when *p *< 0.05 and assigned to six expression profiles by using the following criteria for each protein: (i) protein up-regulated in PR2 profile if the result of the formula PR2 (MEP - MSD)/SP06081 (MEP + MSD) was ≥1.5; and (ii) protein down-regulated in PR2 profile if PR2 (MEP + MSD)/SP06081 (MEP - MSD) was ≤0.67. Proteins whose interval overlapped or did not satisfy these criteria were defined as constant.

### RNA Isolation and Real-Time RT-PCR Analysis

Endogenous mRNA levels of selected genes were measured by a two-step real-time RT-PCR analysis with an ABI 7500 Real-Time PCR System (Applied Biosystems, USA) using Power SYBR^® ^Green PCR Master Mix (Applied Biosystems) and gene-specific primers (see Additional file 1: Table S1). Briefly, RNA was isolated using TRIzol Reagent (Invitrogen). The quality and the integrity of the RNA samples were evaluated by absorbance measurements and agarose electrophoresis (see Additional file 1: Fig. S3). Control (RT-minus) reaction, which includes all components for RT-PCR except for the reverse transcriptase enzyme, was performed to eliminate the contamination of genomic DNA (see Additional file l: Fig. S4). The RT-PCR products were sequenced to confirm their identities. A high-capacity cDNA archive kit (Applied Biosystems) was used to retrotranscribe 2 μg of total RNA in a final volume of 100 μL of water according to the manufacturer's procedure. Then, 5 μL of the cDNA was mixed with 25 μL of Power SYBR green PCR master mix and each primer with optimizing concentration (see Additional file 1: Table S1) in a final volume of 50 μL. PCR was performed under the following conditions: 2 min at 50°C and 10 min at 95°C, followed by 40 cycles of 30 s at 95°C and 1 min at 60°C. After the final cycle, melt curve data were obtained using an additional stage of dissociation. The specificity of the real-time RT-PCR reactions was determined by melt curve analysis of the amplified products (see Additional file 1: Fig. S5). The relative quantification method (delta-delta threshold cycle) was used to evaluate quantitative variation between replicates examined. Real-time PCR was monitored and analyzed by the sequence detection system version 1.4 (Applied Biosystems), and relative expression levels were normalized to mRNA of the principal sigma factor *sigA*.

## List of abbreviations

LC-MS/MS: liquid chromatography-tandem mass spectrometry; pI: isoelectric point; Mw: molecular weight; RT-PCR: reverse transcriptase polymerase chain reaction; emPAI: exponentially modified protein abundance index; GO: gene ontology; TCA: tricarboxylic acid; PFK: 6-phosphofructokinase; PK: pyruvate kinase; GAPDH: glyceraldehyde 3-phosphate dehydrogenase; ABP: ABC transporter ATP-binding protein; MMSAD: methylmalonate semialdehyde dehydrogenase (acylating); GS: glutamine synthetase; ACAT: acetyl-CoA acetyltransferase; MHM: 5-methyltetrahydropteroyltriglutamate-homocysteine methyltransferase; CS: citrate synthase; SCS: succinyl-CoA synthetase subunit alpha; UPS: uroporphyrinogen III synthetase; AhpC: alkyl hydroperoxide reductase; TSA: thiol-specific antioxidant; SAM: S-adenosylmethionine; NADPH: reduced form of nicotinamide adenine dinucleotide phosphate; ATP: adenosine-triphosphate; PTCSRR: putative two-component system response regulator; MASPP: membrane-associated phospholipid phosphatase; DTT: dithiothreitol; ACN: acetonitrile; HPLC: high-pressure liquid chromatography; SC: spectral count; OP: observable peptide; MEP: peptides mean emPAI; MSD: mean square deviation.

## Competing interests

The authors declare that they have no competing interests.

## Authors' contributions

YSL and XZD carried out the proteomic studies, analyzed the data, and participated in the design of the experiments; YSL drafted the manuscript; FH, WPL, and YT participated in sample collection and preparation; YSL and FH carried out the real-time RT-PCR experiment; YSL, YT, and FH carried out the physiological experiments and HPLC analysis of the spinosad yield; SYH participated in the identification of proteins; YJS participated in the study and helped to draft the manuscript; and LQX and XZD supervised this project. All authors read and approved the final manuscript.

## Supplementary Material

Additional file 1 **Information about the spinosad production and Real-time RT-PCR of *S. spinosa *SP06081 and PR2**. This file provides information on the spinosad production of *Saccharopolyspora spinosa *SP06081 and PR2 under different media and oxygen supply conditions (see Figs. S1 and S2). In addition, it presents the correlative data from Real-time RT-PCR (see Figs. S3~S5 and Table S1): eg. integrity detection of the RNA samples and dissociation curve of amplified products of selected genes. Click here for file

Additional file 2 **The identified proteins from the SP06081 strain by LC-MS/MS**. This file provides information on the identified proteins from the SP06081 strain (see Table S2): eg. Swiss-Prot accession number, SEQUEST score, sequence coverage and peptides from all six proteomics run. Functional categories of the identified proteins were elucidated by Gene Ontology (GO) classification (http://www.uniprot.org/). Proteins labeled with asterisk are the share proteins with the PR2 strain. Click here for file

Additional file 3 **The identified proteins from the PR2 strain by LC-MS/MS**. This file provides information about the identified proteins from the PR2 strain (see Table S3): eg. Swiss-Prot accession number, SEQUEST score, sequence coverage and peptides from all six proteomics run. Functional categories of the identified proteins were elucidated by Gene Ontology (GO) classification (http://www.uniprot.org/). Proteins labeled with asterisk are the share proteins with the SP06081 strain. Click here for file

Additional file 4 **Summary of the proteins only expressed in the SP06081 or PR2 strain during RG1 in SM**. This file provides Information on the exclusively identified proteins from the SP06081 and PR2 strain during RG1 in SM (see Tables S4 and S5): eg. NCBI accession number, SEQUEST score, sequence coverage and emPAI values. Functional categories of the identified proteins were elucidated by Gene Ontology (GO) classification http://www.uniprot.org/. Click here for file

Additional file 5 **The proteins significantly down- or up-regulated in the PR2 strain compared with the SP06081 strain during RG1 in SM**. This file provides Information on the proteins significantly down- or up-regulated in the PR2 strain compared with the SP06081 strain during RG1 in SM (see Table S6): eg. NCBI accession number, SEQUEST score, sequence coverage and relative fold change in PR2/SP06081. Differences in protein abundance were evaluated by *t*-test, "D" and "I" indicated decrease and increase of the protein abundance in the PR2 strain in significant manner (*p *<*0.05*) compared to the SP06081 strain, respectively. Click here for file
