# Supplementary material for: Comparative Proteomic Analysis of saccharopolyspora spinosa SP06081 and PR2 strains reveals the differentially expressed proteins correlated with the increase of spinosad yield
Source: Proteome Sci. 2011 Jul 16;9:40. doi: 10.1186/1477-5956-9-40 (PMC3149565; doi:10.1186/1477-5956-9-40)
Supplement: Additional file 1 — Information about the spinosad production and Real-time RT-PCR of S. spinosa SP06081 and PR2. This file provides information on the spinosad production of Saccharopolyspora spinosa SP06081 and PR2 under different media and oxygen supply conditions (see Figs. S1 and S2). In addition, it presents the correlative data from Real-time RT-PCR (see Figs. S3~S5 and Table S1): eg. integrity detection of the RNA samples and dissociation curve of amplified products of selected genes. [file 1477-5956-9-40-S1.DOC]

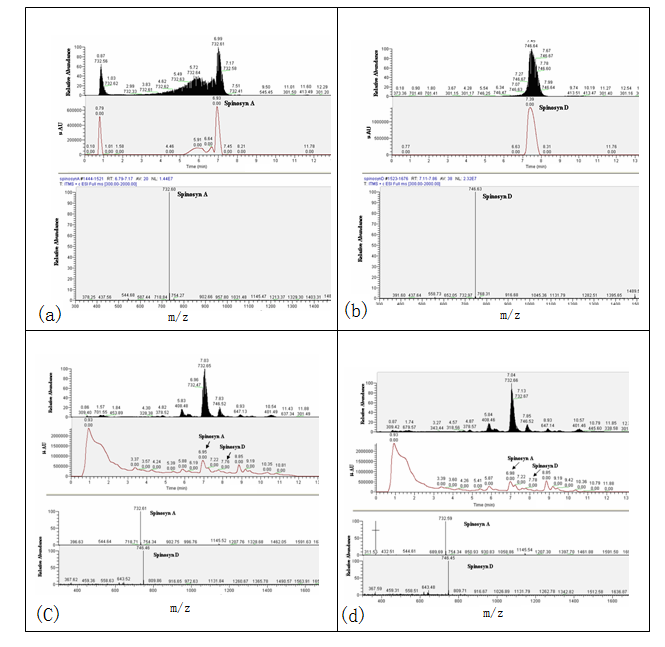


**Figure S1 Detection of the spinosad yields of *Saccharopolyspora spinosa* SP06081 and PR2 in SM by LTQ mass spectrometer.**

Methanolic extract of seed culture from *S. spinosa* SP06081 and PR2 after 9 d culture in seed medium were analyzed by LC-MS/MS. (a) LTQ mass spectrum of standard spinosyn A (concentration 100mg/L, retention time 6.93, molecular weight 732.6), (b) LTQ mass spectrum of standard spinosyn D (concentration 100mg/L, retention time 7.39, molecular weight 746.63), (c) LTQ mass spectrum of *S. spinosa* PR2 fermentation broth, (d) LTQ mass spectrum of *S. spinosa* SP06081 fermentation broth.

After 48 h culture in seed medium, The cells were transferred into the same medium with 10% inoculum for spinosad production (culture conditions: 30°C, 300 rpm, 300ml flask containing 20 ml medium). Three replicates were performed for each strain. The average yields of spinosad in seed medium from SP06081 and PR2 were 11.5 mg/L and 18.8 mg/L respectively (calculated by the area of chromatographic peak), without significant differences (*t*-test, *P*＞0.05)

**Figure S2 Comparative analysis of the spinosad yields in production medium between *Saccharopolyspora spinosa* SP06081 and PR2 under different oxygen supply conditions.**

HDO: Flasks (300ml) containing 20 ml of the production medium at 30°C, 300 rpm, which means relatively high dissolved oxygen supply. LDO: Flasks (150 ml) containing 20 ml of the production medium at 30°C, 200 rpm, which means relatively low dissolved oxygen supply. Fermentation for spinosad production was run for 9 d in a humidized rotary shaker incubator at 30°C and 80% relative humidity. Six replicates were performed for each strain under HDO or LDO (see methods). Error bars indicate standard error of the mean. Asterisk indicate significantly different between two samples (*t*-test, *P*＜0.01).

After 48 h culture in seed medium, about 10% seed culture was transferred into production medium for spinosad production under different oxygen supplies. Compared to the strain SP06081, PR2 displayed higher spinosad productivity both under high dissolved oxygen supply (HDO) and low dissolved oxygen supply (LDO). The difference was even more significant under LDO. Under HDO, the spinosad yield of PR2 increased about 2.3 fold compared with that of SP06081; Under LDO, the strain SP06081 almost lost the productivity of spinosad while PR2 strain maintained about 20% spinosad yield compared with that under HDO.


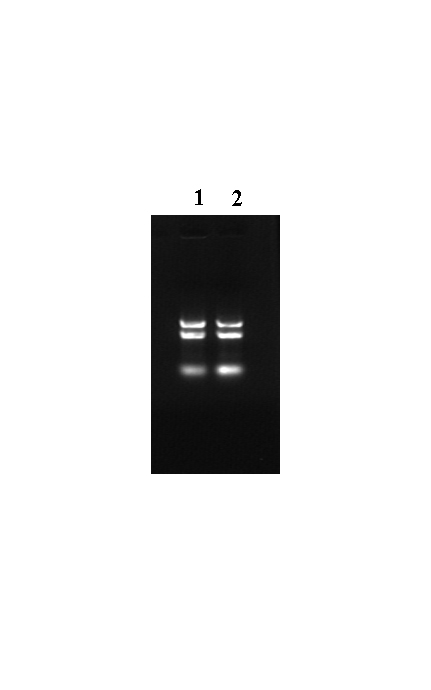


**Figure S3 Integrity detection of the RNA samples extracted from SP06081 ( 1, OD260/OD280＝2.05) and PR2 strains (2, OD260/OD280＝2.01 ) by 1% agarose gel electrophoresis**


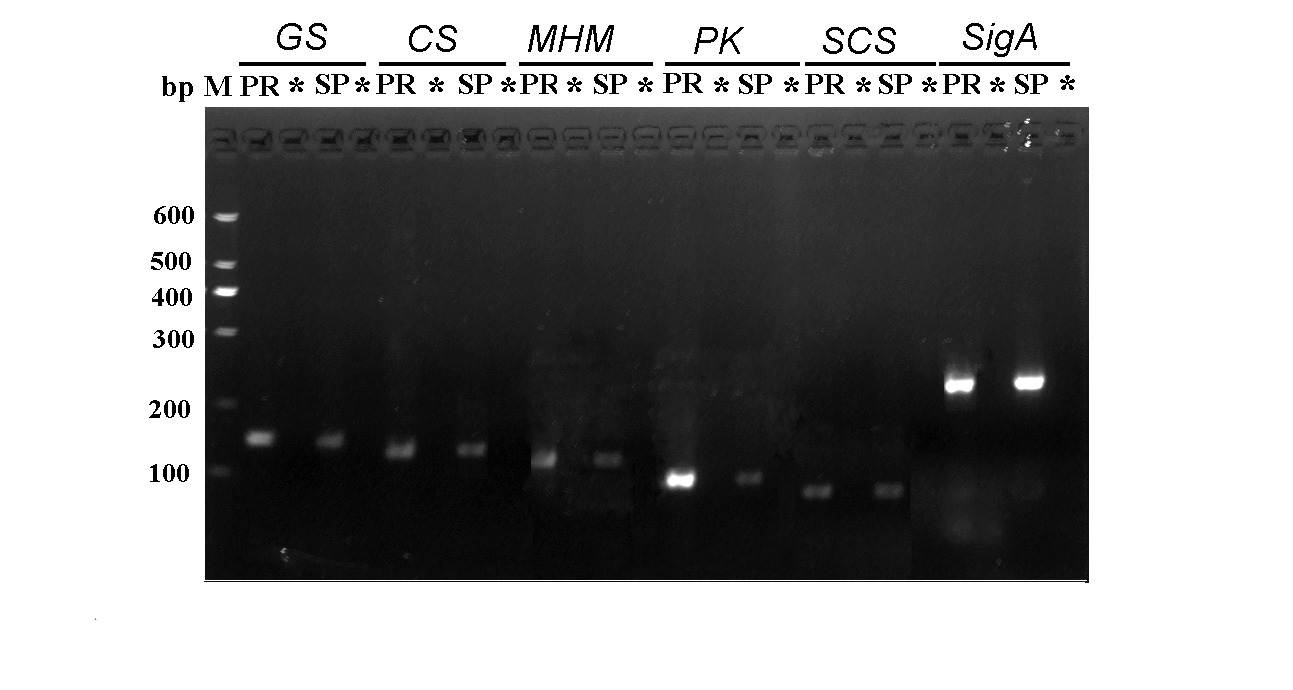


**Figure S4 Transcriptional map of selected genes.** RNA samples extracted from mycelium of strain SP06081 (SP) and PR2 (PR) after 48 h culture were analyzed by RT-PCR using primers specific (see Table S1). Asterisks indicate negative controls (no reverse transcriptase).


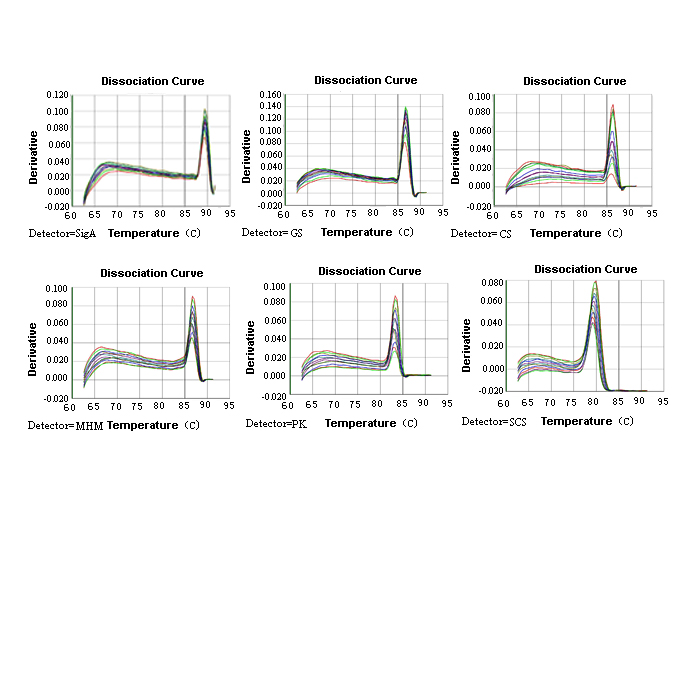


**Figure S5 Dissociation curve of amplified products of selected genes**

| **Table S1 Primers and their optimizing reaction concentration for real-time RT-PCR analysis** | | | |
| --- | --- | --- | --- |
| **Gene** | **Primer name** | **Sequence** | **Reaction concentration** |
| *Sig A* | SigA-F | CTA CCT CAA GCA GAT CGG CAA G | 100nM |
|  | SigA-R | GAT CAG GTC CAG GAA CGC CAT G | 100nM |
| *CS* | CS-F | CCG TTC GAC GCC AAC CAG G | 500nM |
|  | CS-R | CCG CAG GAA GTT CTC CAC CAG | 500nM |
| *GS* | GS-F | ATC GCC GAC ACC GCC TTC TTC | 300nM |
|  | GS-R | CGC CCT TCA TCC GGG TCT TG | 900nM |
| *MHM* | MHM-F | GGG TGC AGT CCT ACG GTT CG | 300nM |
|  | MHM-R | GCC CTT GAC CGG CTT ATC G | 900nM |
| *PK* | PK-F | AGA AGA AGG CGA TCC GCA TA | 500nM |
|  | PK-R | GGT CGG ACG CGA GTT CTG | 500nM |
| *SCS* | SCS-F | CGG ACT ACA TCA AGG CCA ACA | 500nM |
|  | SCS-R | GGT GAA ACC GGC GAC GTA | 500nM |
