# Supplementary material for: Comparative Proteomic Analysis of saccharopolyspora spinosa SP06081 and PR2 strains reveals the differentially expressed proteins correlated with the increase of spinosad yield
Source: Proteome Sci. 2011 Jul 16;9:40. doi: 10.1186/1477-5956-9-40 (PMC3149565; doi:10.1186/1477-5956-9-40)
Supplement: Additional file 5 — The proteins significantly down- or up-regulated in the PR2 strain compared with the SP06081 strain during RG1 in SM. This file provides Information on the proteins significantly down- or up-regulated in the PR2 strain compared with the SP06081 strain during RG1 in SM (see Table S6): eg. NCBI accession number, SEQUEST score, sequence coverage and relative fold change in PR2/SP06081. Differences in protein abundance were evaluated by t-test, "D" and "I" indicated decrease and increase of the protein abundance in the PR2 strain in significant manner (p <0.05) compared to the SP06081 strain, respectively. [file 1477-5956-9-40-S5.DOC]

**Table S6 ：The proteins significantly down- or up-regulated in the PR2 strain compared with the SP06081 strain during RG1 in SM**

| **Accession number ^1)^** | **Protein description** | **Gene ^2)^** | **Main metabolic pathway/Cellular function (category) ^3)^** | **Relative fold change in PR2/SP06081 *(P＜0.05)* ^4)^** | **Sequence coverage (%, PR2/SP06081)** | **SEQUEST**  **Score ^5)^**  **((( PR2/SP06081)** |
| --- | --- | --- | --- | --- | --- | --- |
| gi134097258 | Citrate synthase | CS | Tricarboxylic acid cycle | 0.28 ( D ) | 10/10 | 37/112 |
| gi134103098 | Succinyl-CoA synthetase subunit alpha | SCS | Tricarboxylic acid cycle | 1.9 ( I ) | 17/10 | 169/84 |
| gi134102169 | Pyruvate kinase | PK | Glycolysis | 1.5 ( I ) | 12/7 | 94/74 |
| gi134098286 | 6-Phosphofructokinase | PFK | Glycolysis | 2.3 ( I ) | 10/10 | 74/25 |
| gi134101276 | 5-Methyltetrahydropteroyltriglutamate-homocysteine methyltransferase | MHM | Biosynthetic process | 2.6 ( I ) | 10/10 | 244/121 |
| gi134098206 | Glutamine synthetase | GS | Biosynthetic process | 2.1 ( I ) | 12/7 | 100/54 |
| gi134097670 | Uroporphyrinogen-III synthetase | UPS | Biosynthetic process | 2.2 ( I ) | 10/4 | 87/75 |
| gi134102202 | Anthranilate synthase component I | ASC | Biosynthetic process | 3.0 ( I ) | 6/6 | 45/27 |
| gi134103371 | Uroporphyrinogen III synthase/methyltransferase | UPS/M | Biosynthetic process | 0.50 (D) | 6/6 | 26/ 53 |
| gi134100346 | Aconitate hydratase | ANH | Catabolic process | 1.5 ( I ) | 12/12 | 182/164 |
| gi134102673 | Acetyl-CoA acetyltransferase | ACAT | Catabolic process | 2.2 ( I ) | 9/9 | 51/21 |
| gi134100432 | S-adenosyl-L-homocysteine hydrolase | SAHH | Catabolic process | 0.50 (D) | 5/5 | 36/80 |

**Table S6 ：Continued**

| **Accession number ^1)^** | **Protein description** | **Gene ^2)^** | **Main metabolic pathway/Cellular function (category) ^3)^** | **Relative fold change in PR2/SP06081 *(P＜0.05)* ^4)^** | **Sequence coverage (%, PR2/SP06081)** | **SEQUEST**  **Score ^5)^**  **(P(PR2/SP06081)** |
| --- | --- | --- | --- | --- | --- | --- |
| gi134098221 | Dihydrolipoamide succinyltransferase | DAST | Catabolic process | 0.55 (D) | 9/10 | 116/227 |
| gi134098260 | Asparagine synthase (glutamine-hydrolyzing) | AS | Catabolic process | 0.40 (D) | 6/6 | 38/54 |
| gi134101104 | Enoyl-CoA hydratase/isomerase family protein | ECH/I | Catabolic process | 0.45 (D) | 6/6 | 30/68 |
| gi134098713 | Glyceraldehyde 3-phosphate dehydrogenase | GAPDH | Oxidation reduction | 0.38 (D) | 12/15 | 98/185 |
| gi134098042 | Methylmalonate-semialdehyde dehydrogenase (acylating) | MMSAD | Oxidation reduction | 0.39 (D) | 10/10 | 74/167 |
| gi134097229 | Superoxide dismutase [Fe-Zn] 1 (FeSOD I) | SOD | Oxidation reduction | 0.34 (D) | 13/14 | 175/264 |
| gi134098222 | Dihydrolipoamide dehydrogenase | DLDH | Oxidation reduction | 0.50 (D) | 6/6 | 52/84 |
| gi134097503 | 4-Hydroxyphenylpyruvate dioxygenase | HPDO | Oxidation reduction | 0.33 (D) | 7/5 | 25/45 |
| gi134098120 | AhpC/TSA family protein | AhpC/TSA | Oxidation reduction | 1.7 ( I ) | 22/22 | 139/119 |
| gi32141225 | Oxidoreductase | OR | Oxidation reduction | 2.0 ( I ) | 10/7 | 129/48 |
| gi134103282 | DNA-directed RNA polymerase subunit beta | DDRP1 | Transcription | 2.2 ( I ) | 8/8 | 105/99 |
| gi134103752 | Protein kinase/LuxR family transcriptional regulator | PK/ LuxR | Transcription | 1.9 ( I ) | 4/4 | 31/71 |
| gi134100285 | Hypothetical protein SACE_3750 | HP | Transcription | 1.5 (I) | 10/10 | 256/140 |

**Table S6** ：Continued

| **Accession number ^1)^** | **Protein description** | **Gene ^2)^** | **Main metabolic pathway/Cellular function (category) ^3)^** | **Relative fold change in PR2/SP06081 *(P＜0.05)* ^4)^** | **Sequence coverage (%, PR2/SP06081)** | **SEQUEST**  **Score^5)^**  **(P(PR2/SP06081)** |
| --- | --- | --- | --- | --- | --- | --- |
| gi134103281 | DNA-directed RNA polymerase subunit beta | DDRP2 | Transcription | 0.43 (D) | 5/5 | 43/145 |
| gi134102490 | 30S Ribosomal protein S16 | RPS16 | Translation | 2.3 ( I ) | 18/18 | 76/51 |
| gi134103257 | 50S Ribosomal protein L16 | RPL16 | Translation | 0.55 (D) | 24/24 | 69/99 |
| gi134103298 | 50S Ribosomal protein L1 | RPL1 | Translation | 0.48 (D) | 11/18 | 49/99 |
| gi134103233 | 30S Ribosomal protein S11 | RPS11 | Translation | 0.41 (D) | 20/20 | 26/48 |
| gi134098746 | Putative ABC transporter ATP-binding protein | ABP | Transport | 2.2 ( I ) | 14/5 | 78/58 |
| gi134097449 | Oligopeptide binding protein of ABC transporter | OBP | Transport | 0.53 (D) | 9/9 | 152/176 |
| gi134102718 | F0F1 ATP synthase subunit alpha | FAS | Transport | 0.38 (D) | 7/7 | 78/110 |
| gi134097686 | Peptidase M16-like | PM16 | Proteolysis | 0.49 (D) | 8/8 | 75/76 |
| gi134100136 | Methionine aminopeptidase | MAP | Proteolysis | 2.1 ( I ) | 7/7 | 31/36 |
| gi134097138 | Chaperonin GroEL | GroEL1 | Protein refolding | 2.5 ( I ) | 25/26 | 2138/1478 |
| gi134103145 | 10kD Chaperonin cpn10 | GroES | Protein folding | 1.5 ( I ) | 46/46 | 745/569 |
| gi134103144 | 60kD Chaperonin 2 | GroEL2 | Protein refolding | 0.50 (D) | 16.7 | 60.05 |
| gi134102579 | Histone-like DNA binding protein | HDBP | DNA binding | 3.8 ( I ) | 10/5 | 158/49 |

**Table S6** ：Continued

| **Accession number ^1)^** | **Protein description** | **Gene ^2)^** | **Main metabolic pathway/Cellular function (category) ^3)^** | **Relative fold change in PR2/SP06081 *(P＜0.05)* ^4)^** | **Sequence coverage (%, PR2/SP06081)** | **SEQUEST**  **Score ^5)^**  **(P(PR2/SP06081)** |
| --- | --- | --- | --- | --- | --- | --- |
| gi134103043 | Putative magnesium or manganese-dependent protein phosphatase | MDPP | Phosphoprotein phosphatase activity | 0.50 (D) | 6/6 | 26/27 |
| gi134098318 | Protein recA | RecA | SOS response | 0.44 (D) | 6/6 | 52/58 |
| gi134098451 | Ornithine-oxo-acid transaminase | OAT | Pyridoxal phosphate binding | 0.48 (D) | 9/9 | 38/81 |
| gi134097037 | ABC transporter ATP-binding protein | ABP | ATPase activity | 2.1 ( I ) | 11/11 | 31/14 |
| gi134103514 | Phosphate transport system regulator PhoU-related protein | PTSRP | No description | 0.30 (D) | 13/16 | 19/66 |
| 1) Protein database accession numbers were obtained on NCBI database. | | | | | | |
| 2) Gene product acronym | | | | | | |
| 3) Functional categories of the identified proteins were elucidated by Gene Ontology classification (http://www.uniprot.org/) | | | | | | |
| 4) Differences in protein abundance were evaluated by *t*-test, “D” and “I” indicated decrease and increase of the protein abundance in the PR2 strain in significant manner ( *p＜0.05 )* compared to the SP06081 strain, respectively. | | | | | | |
| 5) The Score is a sum of the individual peptide scores from all six proteomics run (see Additional file 2 and file 3: Tables S2 and S3). | | | | | | |
